# Altered T Cell Memory and Effector Cell Development in Chronic Lymphatic Filarial Infection That Is Independent of Persistent Parasite Antigen

**DOI:** 10.1371/journal.pone.0019197

**Published:** 2011-04-29

**Authors:** Cathy Steel, Thomas B. Nutman

**Affiliations:** Laboratory of Parasitic Diseases, National Institutes of Health, Bethesda, Maryland, United States of America; New England Biolabs, United States of America

## Abstract

Chronic lymphatic filarial (LF) infection is associated with suppression of parasite-specific T cell responses that persist even following elimination of infection. While several mechanisms have been implicated in mediating this T cell specific downregulation, a role for alterations in the homeostasis of T effector and memory cell populations has not been explored. Using multiparameter flow cytometry, we investigated the role of persistent filarial infection on the maintenance of T cell memory in patients from the filarial-endemic Cook Islands. Compared to filarial-uninfected endemic normals (EN), microfilaria (mf) positive infected patients (Inf) had a reduced CD4 central memory (T_CM_) compartment. In addition, Inf patients tended to have more effector memory cells (T_EM_) and fewer effector cells (T_EFF_) than did ENs giving significantly smaller T_EFF_ ∶ T_EM_ ratios. These contracted T_CM_ and T_EFF_ populations were still evident in patients previously mf+ who had cleared their infection (CLInf). Moreover, the density of IL-7Rα, necessary for T memory cell maintenance (but decreased in T effector cells), was significantly higher on memory cells of Inf and CLInf patients, although there was no evidence for decreased IL-7 or increased soluble IL7-Rα, both possible mechanisms for signaling defects in memory cells. However, effector cells that were present in Inf and CLInf patients had lower percentages of HLA-DR suggesting impaired function. These changes in T cell populations appear to reflect chronicity of infection, as filarial-infected children, despite the presence of active infection, did not show alterations in the frequencies of these T cell phenotypes. These data indicate that filarial-infected patients have contracted T_CM_ compartments and a defect in effector cell development, defects that persist even following clearance of infection. The fact that these global changes in memory and effector cell compartments do not yet occur in infected children makes early treatment of LF even more crucial.

## Introduction

Lymphatic filariasis (LF) is a chronic helminth infection that is associated with a profound parasite-specific T cell hyporesponsiveness in individuals with patent infection. Mechanisms underlying this diminished antigen (Ag)-specific T cell response have included: 1) increased production of IL-10 and/or the expansion of IL-10 producing CD4^+^ T cells [1], [2], [3], [4]; 2) *in utero* exposure to the parasite [5], [6] 3) altered Ag presenting cell function [7], [8]; and 4) apoptosis of Ag-activated cells [9]. Furthermore, the increased expression of CTLA-4 [10], [11], PD-1 [10], and Cbl-b [12] as well as modulation of the host response by secreted parasite molecules [13], [14] have also been postulated to play a role in mediating the modulation of T cell responses seen in LF.

The attenuated parasite-specific responses seen in patients with chronic helminth infections have been demonstrated to extend to bystander antigens and to non-filarial infections. Human studies have shown that LF can alter responses to malaria [15], *Mycobacterium tuberculosis* [16] and HIV [17]. In addition, the presence of schistosome [18] or geohelminth [19] infections has been found to alter allergic responses in children. Of even more broad-reaching importance is the impaired response to parenterally- [20], [21] and orally- [22] administered vaccines seen in those with intestinal or tissue invasive helminth infections.

The mechanisms underlying this bystander suppression in chronic helminth infection remain unclear, although it is probable that some of this suppression is due to the filaria-specific mechanisms mentioned above. However, one area that has not been examined is the role that effector and memory T cells may play. Indeed, the majority of studies on effector and memory T cell populations have been conducted in viral infections [23], [24], [25], [26], [27], [28], [29], [30] and intracellular parasitic infections [31], [32], [33], [34], [35] where clear alterations in the function and phenotype of these T cells have been associated with chronic infection. These types of studies have not generally been extended to extracellular parasites (e.g. helminth infections), though a single study in mice with a GI helminth showed a persistence of memory CD4^+^ T cells following sterile cure despite the absence of chronic infection [36].

Using recent advances in flow cytometry and the identification of phenotypic markers that identify specific T cell populations, we studied the effects of chronic filarial infection on effector and memory phenotypes in patients. The association of markers that define memory and effector T cell populations, a selection of which are described below for the current study, has been refined based on data from animal studies [26], [30], [36] and from the study of chronic viral infections in humans [37], [38], [39], [40], [41]. Characterization of these T cell populations is often based on the expression of CCR7, a chemokine receptor important for homing to secondary lymphoid organs, and that of the costimulatory molecule CD27. Both markers are found on CD45RA^+^ naïve cells and long-lived CD45RA^−^ central memory cells, the latter known to proliferate and produce IL-2, displaying effector function only following secondary antigen stimulation. In contrast, the absence of CCR7 characterizes effector memory cells with shorter lifespans, but with the ability to home to sites of inflammation thereby exhibiting immediate effector function [42]. For the purposes of this study, CD45RA^+^ and CD45RA^−^ effector memory cells were also defined as absent for CD27 expression, a subset similar to the late or fully differentiated effector memory cells described previously [43], [44]. The expression of the chemokine receptor CCR5, present on effector site or effector site seeking cells and downregulated following activation [45], [46] was also used to define effector memory cells [26]. Finally, of most importance to the homeostatic maintenance [47], [48] and survival [49], [50] of memory cells is the cytokine IL-7 and its receptor, IL-7Rα (CD127); therefore, in this study, the expression of IL-7Rα was used to define both stable central and effector memory cell populations.

The patient population in this study was from an LF-endemic island in the South Pacific. This population had been studied longitudinally over an 18 year period with extensive collection of clinical, parasitological and immunological data [51], [52], [53], [54]. Previous work demonstrated that chronically infected individuals had suppressed parasite-specific T cell proliferation and cytokine responses, and moreover, this suppression continued even after the filarial infection (and parasite Ag) had been eliminated [53]. The present study extends these observations to include alterations in effector and memory phenotypes associated with LF, alterations (once established) that remain despite clearance of infection.

## Materials and Methods

### Ethics Statement

Both studies were approved by the NIAID Institutional Review Board and informed written consent was obtained from all adult subjects; consent from non-adult subjects were obtained through both verbal assent and written consent from the subjects' legal guardian.

### Patient Population

The study population was comprised of 29 permanent residents of the island of Mauke in the Cook Islands, a region endemic for the filarial parasite *Wuchereria bancrofti*. These individuals were part of a larger assessment of filarial infection of the island population over the course of an 18 year period with 2 primary assessments (1975 and 1992; [51], [52], [53], [54]). A subset of those previously evaluated at both timepoints was used for the current study (Table 1). All subjects had been evaluated for clinical (history, physical examination, and complete blood count), parasitologic (filtration of 1 ml of blood through a Nuclepore™ 3 µm filter to quantify microfilariae) and immunologic parameters during both study periods. In addition, serum samples from both 1975 and 1992 were tested for the presence of circulating filarial antigen (CAg; TropBioPty Ltd., Townsville, Australia) that serves as a marker for the presence of adult worms and, therefore, as an indication of an active infection. A group of age and gender matched children was studied separately. The present study utilized cells cryopreserved in1992 and stored in liquid nitrogen until used.

**Table 1:** Patient Groups.

GROUP	1975/1992 INFECTION STATUS^a^	MALE/FEMALE	AGE RANGE (MEAN)
Endemic Normals (EN)	Negative/Negative	1/5	44–72 (56)
Infected (Inf)	Positive/Positive	4/1	27–67 (56)
Cleared Infected (CLInf)	Positive/Negative	4/2	25–66 (45)
Uninfected Children (ENCh)	N/A/Negative	2/4	7–12 (10)
Infected Children (InfCh)	N/A/Positive	2/4	6–12 (10)

Patient groups from the filarial-endemic island of Mauke in the Cook Islands. Filarial infection status was determined at two timepoints (1975 and 1992); age is shown as the range and mean for each group of patients.

a All patients positive for infection were microfilaremic; negative status indicates a negative test for circulating antigen (CAg) and microfilaremia.

N/A – Not applicable.

### Fluorochrome Antibody Reagents

All fluorochromes were first titrated and negative gates for each were assigned using a “fluorochromes minus one (FMO)” approach [55]. The following antibodies (Ab) were used for each sample: from BD Biosciences (San Jose, CA) – CD3 Pacific Blue, CD4 AmCyan, CD45RA PE-Cy5, CCR7 PE, CD69 FITC, CCR5 APC; from BioLegend (San Diego, CA) – HLA-DR Alexa Fluor 700; from eBioscience (San Diego, CA) – IL-7Rα (CD127) PE-Cy7, CD27 APC-Alexa Fluor 750; and from Molecular Probes (Eugene, OR) – CD8 Qdot 605, Live/Dead Cell Stain Kit UV.

### Cell Preparation

Frozen PBMCs were washed, counted with trypan blue, and plated in 6-well plates at a concentration of 1–1.5×10^6^ cells/ml in RPMI/10% FCS. Cells were incubated at 37°C, 5% CO_2_ for 18 hrs. Prior to harvesting, deoxyribonuclease I (Sigma, St. Louis, MO) was added to the wells for 5 min, 37°C, to prevent clumping. Cells were then washed and resuspended in PBS and stained with the viability dye at RT for 30 min. in the dark. Cells were washed in PBS/1% BSA, blocked with human Ig (Sigma) at RT for 20 min., and subsequently placed into V-bottom plates with fluorochromes for 30 min. on ice in the dark. After labeling, cells were washed, resuspended in PBS/1% BSA, transferred to tubes, and placed on ice in a covered container for immediate flow cytometry.

### Flow Cytometry Analysis

**Figure 1:**
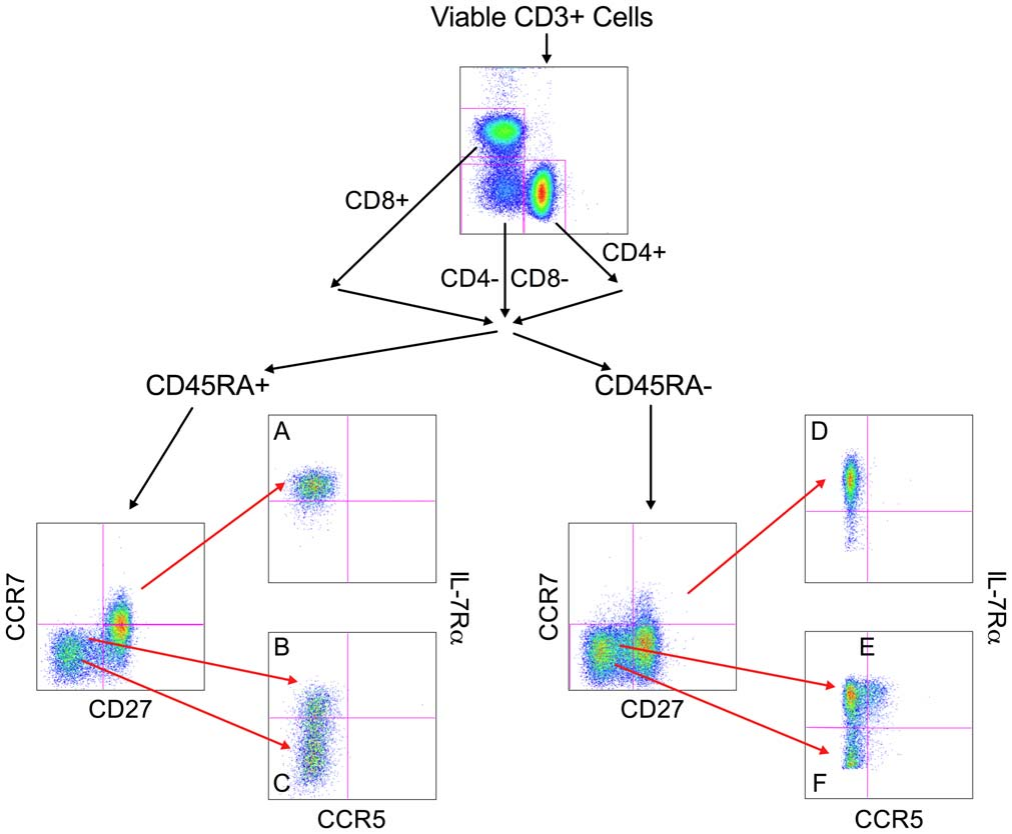
Gating strategy for the 6 major T cell populations beginning with viable CD3^+^ lymphocytes. CD4^+^, CD8^+^, and CD4^−^CD8^−^ cells were separated into CD45RA^+^ and CD45RA^−^ subsets which were subsequently divided into the 6 T cell phenotypes: **CD45RA^+^ cells:** A – Naïve cells (CD45RA^+^CCR7^+^CD27^+^IL-7Rα^+^CCR5^−^); B – T effector memory RA^+^ (T_EMRA_^+^) cells (CD45RA^+^CCR7^−^CD27^−^IL-7Rα^+^CCR5^−^); C – T terminal effector (T_TermEff_) cells (CD45RA^+^CCR7^−^CD27^−^IL-7Rα^−^CCR5^−^); **CD45RA- cells:** D – T central memory (T_CM_) cells (CD45RA^−^CCR7^+^CD27^+^IL-7Rα^+^CCR5^−^); E – T effector memory (T_EM_) cells (CD45RA^−^CCR7^−^CD27^−^IL-7Rα^+^CCR5^+/−^); F – T effector (T_EFF_) cells (CD45RA^−^CCR7^−^CD27^−^IL-7Rα^−^CCR5^−^).

Fluorochrome compensation was accomplished using CompBeads (BD Biosciences) for each Ab. Approximately 200,000 events for each sample were collected on an LSRII flow cytometer (BD Biosciences) and data were analyzed using FlowJo (version 9.0.2; Tree Star, Ashland, OR). Prior to selecting T cell populations, a live cell gate was established after which cells were gated on CD3^+^ cells followed by separation into CD4^+^, CD8^+^, and CD4^−^CD8^−^ populations. To define the various memory and effector subsets, cells were subsequently divided into CD45RA^+^ and CD45RA^−^ populations and analyzed for the expression of CD27 versus CCR7 followed by the expression of IL-7Rα versus CCR5. Figure 1 diagrams the gating strategy.

### T cell Populations

Following division into CD3^+^CD4^+^, CD3^+^CD8^+^, and CD3^+^CD4^−^CD8^−^ populations, the following combinations of markers were used to define the naïve, effector and memory subpopulations of T cells (Fig. 1; letters below match the corresponding letters in the figure). CD45RA^+^ populations were defined as follows: A) Naïve - CD45RA^+^CCR7^+^CD27^+^IL-7Rα^+^CCR5^−^; B) T effector memory RA^+^ (T_EMRA_^+^) - CD45RA^+^CCR7^−^CD27^−^IL-7Rα^+^CCR5^−^; and C) Terminal Effector cells (T_Term Eff_) - CD45RA^+^CCR7^−^CD27^−^IL-7Rα^−^CCR5^−^. CD45RA^−^ populations were defined as follows: D) T Central memory (T_CM_) - CD45RA^−^CCR7^+^CD27^+^IL-7Rα^+^CCR5^−^; E) T effector memory (T_EM_) - CD45RA^−^CCR7^−^CD27^−^IL-7Rα^+^CCR5^+/−^; and F) T Effector cells (T_EFF_) - CD45RA^−^CCR7^−^CD27^−^IL-7Rα^−^CCR5^−^.

Effector memory and effector populations (both CD45RA^+^ and CD45RA^−^) were then assessed for the presence of the activation markers HLA-DR and CD69. For the purposes of this study, the phenotypes of T_EMRA_^+^ and T_EM_ cells as described above would predominantly define fully differentiated memory cells that are CD27^−^ but IL-7Rα^+^. In addition to the populations defined above, there were also transitional effector memory populations in both the CD45RA^+^ and CD45RA^−^ compartments that differed from the T_EMRA_^+^ and T_EM_ cells based upon their expression of CD27 and IL-7Rα.

### IL-7/Soluble CD127 (sCD127) Assays

Concentrations of IL-7 in patient sera were determined using the IL-7 Quantikine High Sensitivity Elisa according to the manufacturer's instructions (R&D Systems, Inc., Minneapolis, MN). Measurement of the soluble IL-7Rα (sCD127) was modified from a technique recently described [56]. For this assay, 10 µg of goat anti-human IL-7Rα polyclonal Ab (R&D Systems) was covalently coupled to 1×10^6^ carboxylated-modified fluorescent beads (Luminex, Austin, TX). Sera was diluted 1∶5 in assay diluent consisting of PBS, 1% BSA, 0.05% Tween 20, 0.05% NaN_3_ and 9 mg/ml EDTA and then added to 5000 Ab-coupled beads for 2 hr at RT. Plates were washed with PBS/0.05% Tween 20 and sCD127 was detected with biotinylated mouse anti-human CD127 (1∶500; BD Biosciences) for 1 hr at RT followed by streptavidin PE (Millipore Corporation, Billerica, MA) for 15 minutes. All incubations were done in CoStar (Corning, Lowell, MA) 3357 V-bottom plates covered in foil on a shaking platform. Samples were analyzed on the Bio-Plex 200 System (Bio-Rad, Hercules, CA) and sCD127 levels were determined from a standardized curve generated using recombinant human CD127 Fc Chimera (R&D Systems).

### Statistical Analyses

Analysis of the differences between patient groups was accomplished with GraphPad Prism version 5.0 (GraphPad, San Diego, CA). A two-tailed Mann-Whitney U test with a 95% confidence interval was used to compare groups and a p-value≤0.05 was considered significant. The Spearman Rank Test was used to determine correlations between parameters.

## Results

### EN vs Inf vs CLInf Subjects

**Figure 2:**
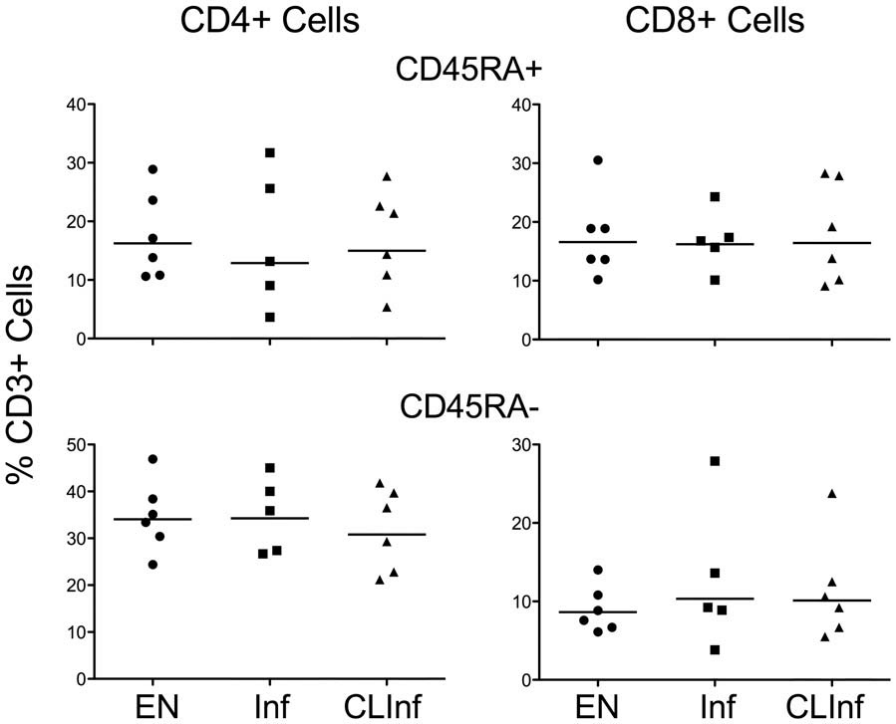
Total CD45RA^+^ and CD45RA^−^ cells. Data for CD45RA^+^ (top panels) and CD45RA^−^ (bottom panels) cells are expressed as a percent of viable CD3^+^ cells in endemic normals (EN), filarial-infected patients (Inf), and in patients who had cleared their filarial infection (CLInf) for CD4^+^ cells (left panels) and CD8^+^ cells (right panels). Each point represents an individual patient and horizontal lines represent geometric means.

Because the major objectives of this study were to assess the consequences of a chronic helminth infection on effector and memory cell populations and to understand whether the clearance of lingering Ag alters these populations, a comparison was made between endemic normal (EN) individuals (never infected) to those who had clear evidence of long-standing, active filarial infection at both time periods (Inf). This was contrasted to those who had been actively infected in 1975, but who had cleared their infection in the intervening 18 years (CLInf). Initial analysis of CD3^+^ cells for the EN, Inf, and CLInf groups showed no differences among the groups in the percentages of either CD45RA^+^ or CD45RA^−^ cells within the CD3^+^ population (Fig. 2). Therefore, any differences observed in subsequent analyses were not a result of differences within these two major CD3^+^ cell compartments. There were also no significant differences in the percentage of naïve T cells (defined as CD45RA^+^CCR7^+^CD27^+^IL-7Rα^+^CCR5^−^) among the groups (data not shown), although there was an increase in the relative size of the naïve compartment in the EN group compared to either the Inf or CLInf groups, particularly for CD4^+^ cells (Table 2). Not unexpectedly, the largest percentages of CD45RA^+^ cells in any of the 3 groups studied were CCR7^−^ (non-naïve).

**Table 2:** Geometric mean % size of each T cell subset relative to all 6 cell phenotypes.

	CD45RA^+^			CD45RA^−^		
	Naive	T_EMRA_^+^	T_TermEff_	T_CM_	T_EFF_	T_EM_
CD3^+^CD4^+^						
EN	**14.5** (7.8–28.0)	**2.4** (0.5–4.2)	**1.6** (0.5–4.2)	**7.9** (2.7–15.5)	**5.9** (2.9–9.9)	**63.4** (49.4–75.5)
Inf	**7.6** (2.0–26.3)	**4.5** (1.6–12.4)	**2.4** (1.0–5.9)	**4.1** (2.0–13.5)	**3.7** (2.6–4.5)	**69.7** (53.2–83.8)
CLInf	**5.7** (1.9–13.1)	**2.8** (1.1–20.8)	**1.5** (0.5–7.5)	**3.5** (0.8–9.9)	**5.0** (3.2–7.3)	**75.0** (64.1–82.9)
ENCh	**5.5** (3.1–11.5)	**2.8** (1.6–5.4)	**2.7** (1.2–5.0)	**3.6** (2.2–6.2)	**8.2** (5.8–10.4)	**75.0** (69.3–80.7)
InfCh	**6.2** (3.3–10.4)	**3.2** (1.6–6.1)	**4.1** (2.2–6.7)	**3.9** (1.8–6.3)	**12.0** (7.1–25.1)	**67.4** (58.9–76.6)
CD3^+^CD8^+^						
EN	**7.9** (2.9–18.8)	**6.1** (4.1–9.1)	**29.3** (22.0–39.9)	**0.7** (0.2–1.5)	**28.1** (19.3–36.2)	**23.0** (14.9–35.9)
Inf	**4.5** (0.7–18.3)	**9.6** (6.9–14.8)	**25.8** (17.2–45.9)	**0.5** (0.1–1.5)	**21.9** (15.7–30.3)	**30.5** (16.0–42.7)
CLInf	**4.8** (3.3–9.0)	**8.2** (4.5–12.2)	**30.5** (22.4–39.3)	**0.5** (0.1–3.0)	**19.2** (15.9–25.1)	**34.1** (26.1–40.9)
ENCh	**5.6** (2.2–8.1)	**3.3** (1.1–8.3)	**22.7** (10.5–37.0)	**0.3** (0.1–0.8)	**43.2** (24.7–64.0)	**17.4** (8.3–32.0)
InfCh	**8.8** (4.6–15.2)	**3.2** (1.4–7.4)	**15.5** (8.3–34.1)	**0.6** (0.4–0.9)	**45.3** (30.7–65.9)	**20.7** (13.1–37.2)
CD3^+^CD4^−^CD8^−^						
EN	**0.6** (0.1–12.1)	**10.8** (4.9–17.2)	**39.9** (26.7–52.6)	**0.2** (0.0–1.4)	**13.8** (11.3–19.2)	**29.4** (19.1–40.3)
Inf	**0.5** (0.1–3.0)	**27.3** (20.3–36.7)	**27.9** (23.6–36.9)	**0.1** (0.0–0.4)	**10.5** (7.0–22.2)	**31.0** (25.9–39.8)
CLInf	**0.6** (0.1–1.4)	**9.5** (3.0–17.6)	**44.5** (30.4–62.3)	**0.1** (0.0–0.6)	**8.8** (6.9–12.0)	**32.1** (18.0–42.8)
ENCh	**0.5** (0.2–0.7)	**9.5** (3.6–15.6)	**25.0** (13.4–41.5)	**0.02** (0.0–0.1)	**12.4** (6.7–21.5)	**46.7** (31.3–67.3)
InfCh	**0.8** (0.4–1.3)	**7.8** (4.5–13.7)	**24.3** (18.7–36.7)	**0.05** (0.0–0.1)	**13.8** (6.9–37.1)	**46.8** (27.3–65.0)

The percent of each cell population for the CD45RA^+^ cells (including naïve, T_EMRA_^+^, and T_TermEff_; A, B, and C in Fig. 1 respectively) and the CD45RA^−^ cells (including T_CM_, T_EM_, and T_EFF_; D, E, and F in Fig. 1 respectively) was calculated for a given patient and the % contribution of each population relative to the six total populations was then determined. The geometric mean percent of each cell phenotype (compartment) for each patient group was then calculated based upon the percentages derived from all of the patients within a group. Ranges for each category are shown in parentheses.

EN – Endemic Normal; Inf – Filarial-infected; CLInf – Cleared Infected; ENCh – Uninfected Children; InfCh – Filarial-infected Children.

#### Effector and Effector Memory Cells

**Figure 3:**
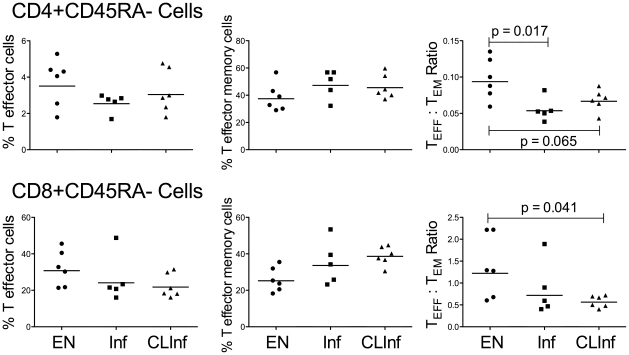
Total T_EFF_ and T_EM_ cells. Data for T_EFF_ (left panels) and T_EM_ (middle panels) cells are expressed as a % of CD4^+^CD45RA^−^ cells (top panels) and CD8^+^CD45RA^−^ cells (bottom panels) in EN, Inf, and CLInf patients. The right panels display the T_EFF_ to T_EM_ ratio for each patient using values derived from percentages of CD45RA^−^ cells for each T cell subset. Each point represents an individual patient and the horizontal lines represent geometric means; the Mann-Whitney U test was used to derive p-values.

**Figure 4:**
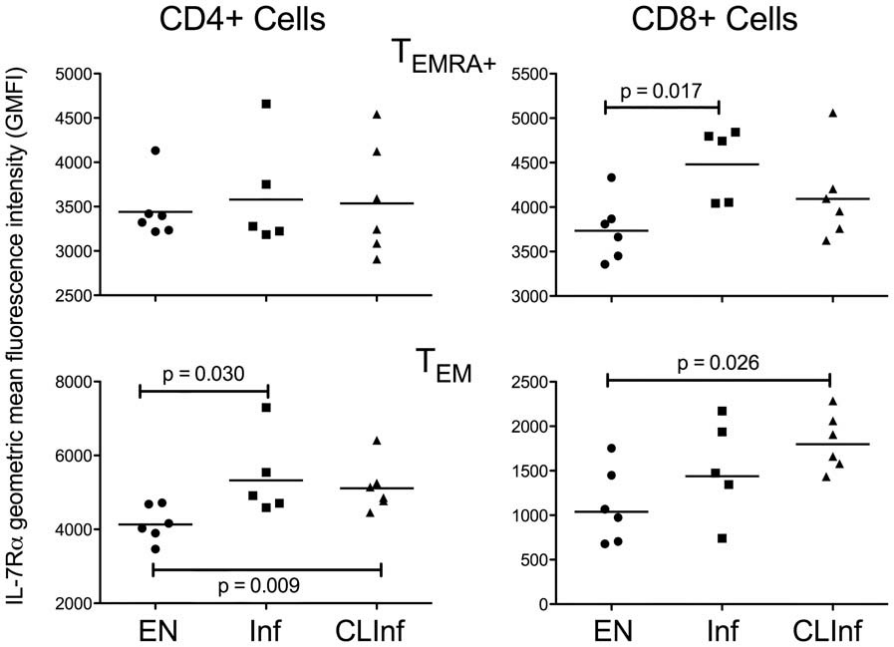
Geometric mean fluorescence intensity (GMFI) of IL7-Rα expression. GMFI of IL-7Rα is shown for EN, Inf, and CLInf patients for T_EMRA_^+^ cells (top panels) and T_EM_ cells (bottom panels) in CD4^+^ cells (left panels) and in CD8^+^ cells (right panels). Each point represents an individual patient and the horizontal lines represent geometric means; the Mann-Whitney U test was used to derive p-values.

Examination of the T_EMRA_^+^, T_TermEff_, T_EM_, and T_EFF_ phenotypes in the 3 patient groups showed that while there were no significant differences in the percentage of T_EFF_ and T_EM_ cells among the groups, the EN group had slightly higher numbers of T_EFF_ but fewer T_EM_ cells in the CD45RA^−^ population than those with active (Inf) or previous but cleared (CLInf) infection (Fig. 3). This is most notable when the T_EFF_ to T_EM_ ratio was calculated for either the CD4^+^ or CD8^+^ cells (Fig. 3; geometric mean [GM] % for CD4^+^ cells = 0.094 [EN] vs 0.054 [Inf, p = 0.017] and 0.067 [CLInf, p = 0.065]; for CD8^+^ cells = 1.22 [EN] vs 0.56 [CLInf, p = 0.041]). Coincidentally, the proportion of the total of the 6 major T cell phenotypes (described in Table 2) that was comprised of either T_EFF_ or T_EM_ cells illustrated that the EN group had a larger T_EFF_ compartment. This contrasted with those with active infection in which the Inf patients had a larger T_EM_ compartment particularly within the CD8^+^ cell population. A similar comparison between the EN and CLInf groups demonstrated that clearance of parasite antigen did not appear to change the size of either the T_EFF_ or T_EM_ compartment from that seen in patients with active infection. In addition, although it did not reach statistical significance, the ratio of T_TermEff_ to T_EMRA_^+^ among CD8^+^CD45RA^+^ cells was higher in the ENs than in the other 2 groups (GM % = 4.8 [EN] vs 2.7 [Inf] and 3.7 [CLInf]). There was, however, a higher geometric mean fluorescence intensity (GMFI) of IL-7Rα (CD127) on effector memory cells in Inf and CLInf patients compared to ENs. For T_EMRA_^+^ cells, this increased expression of IL-7Rα was observed in the CD8^+^ population (EN vs Inf, p = 0.017; Fig. 4), but the disparity in IL-7Rα expression between the EN group compared to either the Inf or CLInf groups was even greater when the CD45RA^−^ T_EM_ compartments were examined (CD4^+^ cells: EN vs Inf [p = 0.030] and CLInf [p = 0.009]; CD8^+^ cells: EN vs CLInf [p = 0.026]).

#### Transitional Memory Cells

In addition to the 6 major T cell phenotypes detailed, there were a number of transitional effector memory cell subtypes found within both the CD45RA^+^ and CD45RA^−^ compartments. The most prevalent of these were CCR7^−^CD27^+^IL-7Rα^+^ with 66–73% found in the CD4^+^CD45RA^+^ compartment. However, there were no differences among the 3 patient groups for this presumed transitional effector memory cell subtype. A second transitional phenotype (CCR7^−^CD27^+^IL-7Rα^−^), a probable effector cell recently activated through the CD70 ligand [57], was noticeably reduced in Inf and CLInf patients within the CD8^+^CD45RA^−^ compartment compared with EN individuals (GM% of CD8^+^CD45RA^−^ cells = 4.1 and 4.2 vs 8.3 respectively; p = 0.015 CLInf vs EN).

#### Central Memory Cells (T_CM_)

Comparison of T_CM_ percentages among the EN, Inf, and CLInf patients demonstrated that EN individuals had an almost 2 fold increase in these CD45RA^−^CCR7^+^CD27^+^IL-7Rα^+^CCR5^−^ cells within the CD4^+^ pool compared to either the Inf or the CLInf subjects (GM% of CD4^+^CD45RA^−^ cells = 4.7 for EN vs 2.8 for Inf or 2.1 for the CLInf). Indeed, the relative contribution of the CD4^+^ T_CM_ compartment was approximately twice as large in the EN group compared to that of the Inf or CLInf group (GM % T_CM_ compartment size = 7.9 [EN] vs 4.1 [Inf] and 3.5 [CLInf]; Table 2). T_CM_ cells did not differ for the CD8^+^ compartment and, unlike what was observed for effector memory cells, the GMFI of IL-7Rα did not differ among the patient groups for either CD4^+^ or CD8^+^ cells.

#### Expression of activation markers

When the surface expression of HLA-DR (Table 3) was assessed on each of the cell populations, EN patients had a higher % of CCR7^−^CD27^−^HLA-DR^+^ cells than did the Inf and CLInf individuals for CD4^+^ T_TermEff_ cells (EN vs Inf, p = 0.018) and T_EFF_ cells (EN vs Inf and CLInf, p = 0.030 and 0.041 respectively). A similar, but not significant, trend was seen for CD8^+^ T_EFF_ cells (EN vs Inf and CLInf, p = 0.052 and 0.065 respectively; Table 3). There were no significant differences in the expression of CD69 among the groups on any of the cell populations studied (data not shown).

**Table 3:** HLA-DR expression on Effector Cells.

	TERMINAL EFFECTOR CELLS	EFFECTOR CELLS
CD4^+^ Cells		
EN	2.6 (1.3–8.2)^a^	3.3 (2.0–4.8)^a^ ^b^
Inf	0.7 (0.3–1.0)	1.6 (1.2–2.5)
CLInf	1.3 (0.4–4.4)	1.9 (1.0–2.5)
ENCh	0.9 (0.2–1.5)	2.6 (2.0–4.0)
InfCh	1.9 (0.6–8.4)	3.9 (2.4–5.8)
CD8^+^ Cells		
EN	21.9 (13.8–31.0)	19.6 (9.0–28.1)
Inf	17.0 (14.9–20.8)	9.5 (5.9–16.1)
CLInf	19.0 (10.7–30.9)	11.2 (6.3–17.3)
ENCh	14.2 (7.7–22.7)	22.4 (12.2–46.9)
InfCh	15.8 (10.4–24.8)	25.3 (21.9–31.5)

Values are the geometric mean (range) of HLA-DR expressing cells for each patient group for CD4^+^ and CD8^+^ effector cells. HLA-DR^+^ expression was determined as a % of CCR7^−^CD27^−^ cells within either the CD45RA^+^ compartment (Terminal Effector Cells) or CD45RA^−^ compartment (Effector Cells).

EN – Endemic Normal; Inf – Filarial-infected; CLInf – Cleared Infected; ENCh – Uninfected Children; InfCh – Filarial-infected Children.

a Indicates a significant difference between EN and Inf.

b Indicates a significant difference between EN and CLInf.

### Infected vs. Uninfected Children

To examine whether the apparent differences in the effector and effector memory compartments observed in adults with long-standing filarial infection occurs with infection of much shorter duration (e.g. young children), assessments of T cell compartments were made between filarial-infected (InfCh) and uninfected (ENCh), age and gender matched children. Despite the finding that children had a higher frequency of CD4^+^CD45RA^+^ cells compared to adults (GM% of CD3^+^CD4^+^ cells = 29.4 [EN] vs 53.8 [ENCh] and 45.9 [InfCh]), the frequency of those that were CD4^+^CD45RA^+^CCR7^+^CD27^+^ was contracted in the children compared to the adults (GM% of CD4^+^CD45RA^+^ cells = 9.6 [EN] vs 3.3 [ENCh] and 3.7 [InfCh]).

**Figure 5:**
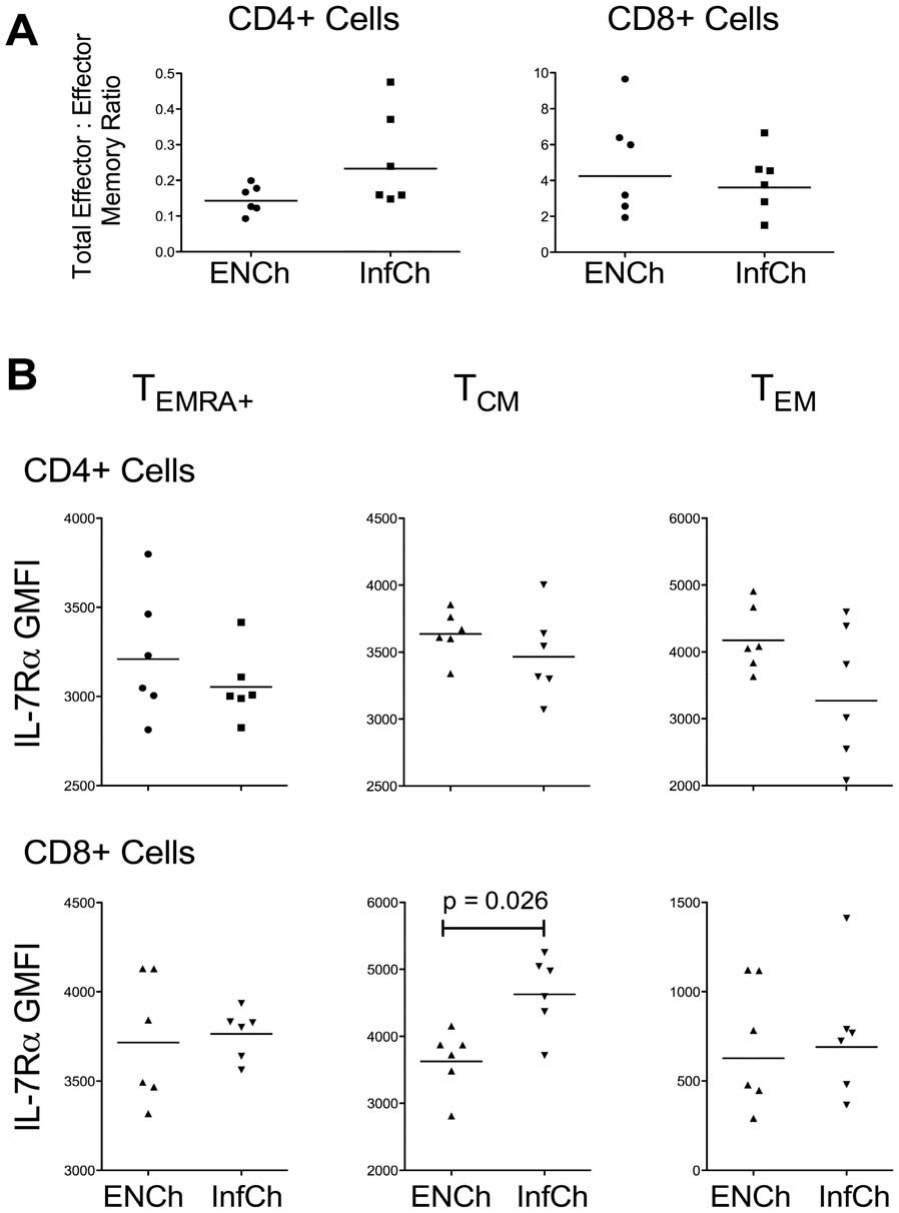
Comparison of endemic normal children (ENCh) and filarial-infected children (InfCh). A) Total effector∶effector memory ratios in CD4^+^ cells (left panel) and CD8^+^ cells (right panel). Values were calculated for each patient using the percentages of either CD45RA^+^ or CD45RA^−^ cells and ratios were derived using the following equation: (% T_TermEff_+% T_EFF_)/(% T_EMRA_^+^+% T_EM_). B) Geometric mean fluorescence intensity (GMFI) of IL-7Rα expression in T_EMRA_^+^ cells (left panels), T_CM_ cells (middle panels), and T_EM_ cells (right panels) for CD4^+^ cells (top panels) and CD8^+^ cells (bottom panels). Each point represents an individual patient and the horizontal lines represent geometric means; the Mann-Whitney U test was used to derive p-values.

The most evident finding was that, unlike that seen for adults, the EN (filarial uninfected) and Inf children did not differ in the composition of the CD4^+^ and CD8^+^ T cell compartments (Fig. 5, Tables 2 and 3). Indeed, most striking was the fact that, unlike in infected adults, the T cell phenotype of infected children included larger compartments comprised of effector T cells, particularly the CD4^+^ T_TermEff_ and T_EFF_ compartments (Table 2). Coupled with the finding of an equivalent total (CD45RA^+^ and CD45RA^−^) effector∶effector memory ratio in InfCh compared to ENCh in both the CD4^+^ and CD8^+^ compartments (Fig. 5A), infection in children was not associated with a shift in effector/effector memory phenotype as seen in adults with filarial infection. Furthermore, there were no differences in IL-7Rα expression on most CD3^+^ cells in InfCh (compared to ENCh) (Fig. 5B) with the exception of CD8^+^ T_CM_ cells (GMFI = 4628 [InfCh] vs 3626 [ENCh], p = 0.026).

### CD4^−^CD8^−^ Cell Population

**Figure 6:**
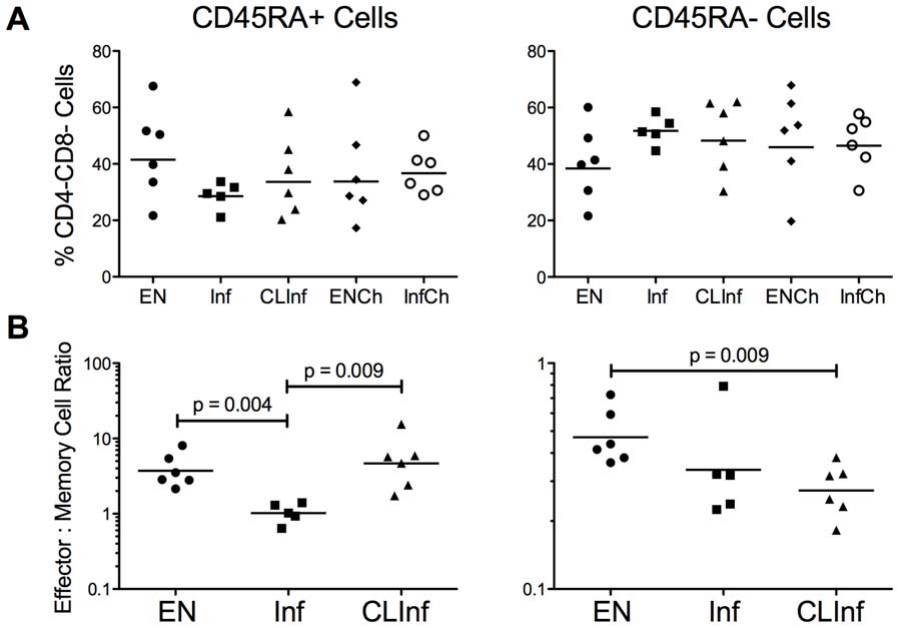
T cell populations in the CD3^+^CD4^−^CD8^−^ compartment. A) Total CD4^−^CD8^−^ cells expressed as a % of CD45RA^+^ cells (left panel) and CD45RA^−^ cells (right panel) in all 5 patient groups. B) Effector∶Effector memory ratios in adult patient groups for CD45RA^+^ cells (% T_TermEff_/% T_EMRA_^+^; left panel) and CD45RA^−^ cells (% T_EFF_/% T_EM_; right panel). Each point represents an individual patient and the horizontal lines represent geometric means; the Mann-Whitney U test was used to derive p-values.

Aside from the classical CD3^+^CD4^+^ and CD3^+^CD8^+^ cells, ∼8.5% (range = 5.8–10.9%) of the viable CD3^+^ cells were CD4^−^CD8^−^ in all patient groups studied. This unusual population appeared to be divided between both CD45RA^+^ and CD45RA^−^ cells (34.7% [range 28.5–41.5] and 45.1% [range 38.4–51.7] respectively; Fig. 6A) and displayed a definitive effector or effector memory phenotype, including a strikingly large contribution (GM = 27.3%) from the CD4^−^CD8^−^ T_EMRA_^+^ compartment in Inf patients (Table 2).

Given the effector and effector memory nature of these CD4^−^CD8^−^ cells, the effector∶effector memory ratios were examined for both the CD45RA^+^ and CD45RA^−^ compartments. Within the CD45RA^+^ cell pool, Inf patients had a significantly lower T_TermEff_ to T_EMRA_^+^ ratio compared with the EN group (p = 0.004) within this double negative population; however, the CLInf group was, for the first time, more similar to ENs, displaying a significantly higher T_TermEff_ to T_EMRA_^+^ ratio than the Inf group (p = 0.009). This latter finding, however, was not seen in the CD4^−^CD8^−^ CD45RA^−^ cells in which both the Inf and CLInf groups had lower T_EFF_ to T_EM_ ratios in comparison to EN patients. There were no differences among the groups in the expression of IL7-Rα, CD69, or HLA-DR on these CD3^+^CD4^−^CD8^−^ cells (data not shown).

### IL-7 and sCD127 Levels in Patient Sera

**Figure 7:**
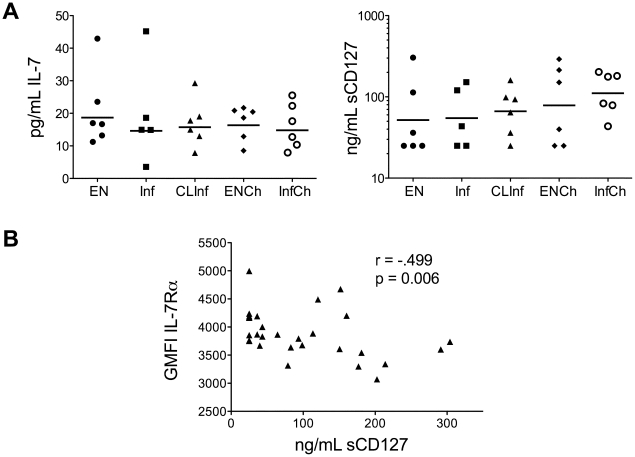
IL-7 and soluble IL-7Rα in patient sera. A) IL-7 (left panel) and soluble IL-7Rα (sCD127; right panel) levels as measured in the sera of patients from all 5 patient groups. B) Correlation between sCD127 levels in the sera and geometric mean fluorescence intensity (GMFI) of IL-7Rα expression on CD4^+^ T_CM_ cells (as measured by flow cytometry).

Because of the importance of IL-7 and sCD127 (the soluble IL-7Rα) in chronic viral infections in humans in maintaining T cell homeostasis [23], we compared the serum levels of IL7 and sCD127 (Fig. 7) in all patients. Examination of IL-7 levels showed a broad range but no differences were seen between any of the groups studied. Similar results were found for levels of sCD127, and there was no relationship between serum levels of IL-7 and the levels of sCD127. Comparable findings were seen when an expanded number of subjects from the Cook Islands (n = 68) were examined (data not shown). There was, however, a negative correlation between the GMFI of IL-7Rα on T_CM_ cells and the levels of sCD127 (Fig. 7b), demonstrating a dynamic between the cell bound and the soluble IL-7 receptor.

## Discussion

Unlike studies of chronic viral infections and the few studies on intracellular parasites (e.g. *Trypanosoma cruzi* [33], [58], *Toxoplasma gondii* [32] and *Leishmania spp.* [35], [59]), data on effector and memory populations in chronic extracellular parasitic infections are lacking. Study of the Ag-specific populations in human helminth infections is hampered by the lack of robust methods to identify Ag-specific cells because of the intrinsic variability in human MHC Class-II recognition of parasite peptides. Moreover, because of the chronicity (and long-lived nature) of these organisms, continuous exposure of the immune system to the parasite's antigens may perturb memory and effector populations, making it difficult to prove unequivocally what occurs in the absence of infection.

Nevertheless, using a group of well-characterized (and longitudinally followed) patients from the Cook Islands, we have been able to identify memory and effector populations with the goal of determining whether the decrease in parasite-specific T cell responses seen in patients with chronic filarial infection [52], [60], [61] can be attributed, at least in part, to an alteration in the effector and memory populations. While most previous studies on filarial infection have examined parasite-specific responses, it has been clearly demonstrated that patients with filarial infections have modulated immune responses to other pathogens [15], [16], [17]. Thus, while we have not yet defined antigen-specific effector and memory populations, because of the lack of methods to mark the filarial-specific cell populations, we have been able to demonstrate alterations in these cell populations *ex vivo* that likely contribute to the Ag-specific T cell hyporesponsiveness seen previously *in vitro* [53].

A limitation of this study was the relatively small size of the patient groups, a limitation based on the paucity of patients within a given study group from whom there were cryopreserved cells. It is also apparent that there was a skewed male∶female ratio among the patient groups. This however reflects the well-known finding that LF is much less common in women of childbearing age. Nevertheless, gender differences in filarial-specific immunological responsiveness have never been seen. In addition, there were no differences in the age ranges among the adult groups studied, a variable that would be more likely to affect infection status and possibly immune responses. Thus, despite these limitations, we were able to show statistically significant differences in memory cell populations in these very well defined patient groups.

The results of this study demonstrated that there were lower numbers of T effector cells in filarial-infected patients in comparison to filarial-uninfected endemic normals not only within the CD4^+^CD45RA^−^ and CD8^+^CD45RA^−^ compartments, but also in CD4^−^CD8^−^ cells (cells thought to contribute to inflammatory responses [62]). Furthermore, a decrease in the population of cells transitioning from a memory to effector state (i.e. CCR7^−^CD27^+^IL7-Rα^−^ cells) was also observed in Inf patients. More importantly, this finding of a reduced effector compartment was seen in those who had cleared the infection and with it, the associated circulating parasite Ag. These data parallel those seen in *in vitro* functional studies on these same patients [53], which demonstrated reduced proliferative and cytokine responses to parasite Ag. In addition, the CD45RA^+^ and CD45RA^−^ effector cells that were identified in Inf and CLInf patients showed a reduced activation state based on their lower expression of HLA-DR, contrasting with findings seen in HIV-1 infected patients [25].

The reduction in the effector compartment in Inf patients might imply that these cells are turning over more rapidly from activation-induced cell death as a result of the constant exposure to circulating parasite antigens; however, this does not explain why the CLInf patients should have a similarly contracted effector compartment. Moreover, the reduced naïve compartment in both Inf and CLInf patients (Table 2) suggests there should be a larger T_EFF_ compartment in these patients because of constant effector cell recruitment from the naïve compartment. One possible explanation for the reduced effector phenotype in Inf and CLInf patients might be the reduction in IL-2 and IFN-γ in these patients [52], [53], cytokines known to be important for reversal of Ag-induced non-responsiveness and conversion of naïve cells to an activated effector state [63].

Another important finding from our study was that filarial infection was associated with an expanded population of effector memory cells that altered the effector∶effector memory ratio. This altered phenotype was widespread, occurring in each of the CD3^+^ populations (CD4^+^, CD8^+^, CD4^−^CD8^−^) and notably, was seen not only in those with active infection but in those who had eliminated the infection (CLInf), suggesting that this may lead to diminished effector cell function. Indeed, it has been hypothesized that chronic Ag stimulation, apparent in older individuals with chronic CMV infection, leads to an increase in senescent dysfunctional cells, primarily within a CD8^+^CD45RA^+^CD27^−^ subset [64]. This population, similar to the T_EMRA_^+^ cells in the current study, was increased in both the Inf and CLInf subjects (Table 2). While these cells could represent those that develop into strong effectors as demonstrated in Mycobacterium infection [65], they could also comprise an accumulating but exhausted CD8^+^ memory cell population with diminished Ag-responsiveness, found more commonly in older individuals [66]. Such immunosenescence has been demonstrated in CD4^+^ cells during *T. cruzi* infection [58]. Further studies of these cell populations for effector function (e.g. granzymes, perforin) will be necessary to elucidate the true nature of these cells.

Unlike T_EM_ cells, the size of the CD4^+^ T_CM_ compartment was reduced by nearly 50% in both Inf and CLInf patients in comparison to EN individuals. This suggests that filarial infection (past or present) is associated with a defect in long-term memory function that may be reflected in the inability to proliferate and produce IL-2 in response to Ag [52], [53]. The importance of a strong T_CM_ compartment is demonstrated by the ability of these cells to persist longer than T_EM_ cells *in vivo*, even in the absence of Ag [67], and to mediate protection because of their high proliferative potential [68]. Indeed, protection against the intracellular parasite *L. major* is mediated not only by short-lived effector cells but also by the long-lived pathogen independent T_CM_ cells [35]. Furthermore, since human T_CM_ and T_EM_ cells do not interconvert [69], the decrease in the numbers of T_CM_ cells (and the increased numbers of T_EM_ cells) cannot be attributed to interconversion.

Surprisingly, data from this study found that not only were there more effector memory cells in Inf and CLInf patients, these cells had a higher mean expression of IL-7Rα (CD127), a finding that differs from what has been seen in chronic viral infections in humans [25], [38], [40] and in mice [70], [71]. Nevertheless, our data support the notion that filarial infection is associated with a diminished ability of effector memory cells to convert to T effectors. Given that IL-7Rα should decrease upon Ag activation [49] and that patient T cells are under constant exposure to parasite Ag, there is an expectation of a larger IL-7Rα^−^ pool. Our data, however, shows just the opposite. There may be several reasons for these differences including: 1) a lack of IL-2 [53] needed for the negative regulation of IL-7Rα [72]; 2) the development of long-term tolerance in cells that are IL-7Rα^hi^ [73]; 3) a slower turnover rate in aged cells [74], given the chronicity of filarial infection and the age of the patients; and 4) a possible signaling defect in the balance between IL-7Rα^hi^ and IL-7Rα^lo^ cells through the interaction of two proteins – GABPα and GF/-1 [75].

Since IL-7 is important for the differentiation and survival of memory cells [32], we examined IL-7 levels in the sera of patients to determine if lower levels in infected patients might be a limiting factor for signaling in memory cells. Our data failed to support this concept. In addition, there was no relationship between the serum levels of IL-7 and the soluble IL-7Rα (sCD127). There were also no differences seen among patient groups in the serum levels of sIL-7Rα suggesting that sCD127 is not preventing the binding of IL-7 to memory cells [23]. There was, however, a negative correlation between sIL-7Rα and the expression of surface IL-7Rα, corroborating the typical dynamic often seen in cell-bound and soluble IL-7R.

Finally, the altered cellular phenotypes seen in adult patients with long-term filarial infection, either past or present, was not seen in young filarial-infected children, despite their lack of T cell proliferation and cytokine responses to parasite Ag ([52], Steel, unpublished). Thus, the question must be asked - are the mechanisms of T cell hyporesponsiveness different in children? Data from this study would suggest they are. More than likely, other immunological mechanisms of immune regulation (e.g. IL-10, APC dysfunction, *in utero* tolerization) may play a more prominent role in children (or in more recent infection). Indeed, murine LCMV studies predict that if Ag is eliminated during the early stages of infection, memory cells can develop normally [30]; therefore, it might be predicted that, while these children have some loss of T cell function, the global defects in the effector and memory compartments seen with long-term infection might be avoided if the filarial infection is eliminated early in life.

In summary, we have demonstrated a change in the nature of effector and memory populations that characterize patients with chronic filarial infection. Coupled with previous studies that have shown a modulation of effector responses to other pathogens [15], [16], [17] and to parenteral and oral vaccinations [20], [21], [22], the data from this study suggests that there are even more broad-reaching consequences of persistent filarial infection that provides additional impetus to treat filarial infections as early in life as possible.
